# Unmet needs and associated factors among patients with melanoma: a mixed-methods study

**DOI:** 10.3389/fpubh.2026.1956448

**Published:** 2026-09-28

**Authors:** Juan Liao, Miao Rao, Yuanyuan Wang, Long Long, YuPing Cai, Rongquan Wei

**Affiliations:** 1 Guangxi Medical University Cancer Hospital, Nanning, China; 2 Department of Intensive Care Medicine, Guangxi Medical University Cancer Hospital, Nanning, China; 3 Department of Bone and Soft Tissue Surgery, Guangxi Medical University Cancer Hospital, Nanning, China; 4 Department of Nursing, Guangxi Medical University Cancer Hospital, Nanning, China

**Keywords:** information needs, melanoma, mixed-methods study, social ecological theory, unmet needs

## Abstract

**Background:**

The burden of melanoma in China is increasing, but the supportive care needs of Chinese patients remain understudied. The applicability of Western findings may be limited by clinical and sociocultural differences. This study aimed to characterize unmet needs among Chinese patients with melanoma and identify associated factors using a mixed-methods approach.

**Methods:**

A convergent parallel mixed-methods study was conducted. Quantitatively, 105 patients completed the Needs Evaluation Questionnaire, and multivariable binary logistic regression was performed to identify associated factors. Qualitatively, semi-structured interviews were conducted with an independent sample of 16 patients. Directed content analysis was guided by the Social Ecological Model. The quantitative and qualitative findings were integrated through a joint analysis.

**Results:**

Information needs were the most frequently reported unmet needs. In the multivariable model, female patients had lower odds of high unmet needs than male patients (*OR =* 0.257, *95%CI*: 0.080–0.830, *p* = 0.023). Internet-based health information use was also associated with lower odds of high unmet needs (*OR* = 0.075, *95%CI*: 0.022–0.257, *p* < 0.001). Nine qualitative themes were identified across individual, family/community, and sociocultural levels. Individual-level factors were most prominent. The integrated findings reflected the interplay of personal vulnerability, social support, healthcare access, information resources, and sociocultural contexts.

**Conclusion:**

Unmet needs, particularly information needs, were prominent among Chinese patients with melanoma and reflected factors across multiple ecological levels. Patient-centered care should prioritize tailored information support and address barriers to healthcare access and supportive resources.

## 1 Background

Melanoma is a highly malignant tumor that originates from melanocytes. It is characterized by strong invasiveness, rapid metastasis, and high mortality. Therefore, it is regarded as the most dangerous type of skin tumor (1). Asian populations have long been considered a low-incidence group. However, recent evidence shows that the disease burden of melanoma in China is changing significantly. Yu et al. (2) analyzed data from the Global Burden of Disease Study 2021. Between 1990 and 2021, the age-standardized incidence rate of melanoma in China increased by 89.2%. The number of patients increased more than threefold, from approximately 3,250 to 13,437. In European and American countries, melanoma mainly occurs in light-skinned populations and is attributed to intermittent sun exposure. In contrast, melanoma in China predominantly comprises acral and mucosal subtypes. Its incidence is closely related to chronic injury and mechanical friction. Furthermore, population aging, rather than UV exposure, has been identified as the primary driver of the increasing disease burden (3–5).

Advances in diagnostic and therapeutic technologies have extended cancer patients’ survival. However, unmet needs for information, psychology, and supportive care persist (6). Chinese patients with melanoma may face multiple unmet needs. Unmet needs are defined as the gap between received care and perceived needs (7, 8). This concept originated in palliative care and has since expanded to tumor supportive care, as unmet needs negatively affect health outcomes. Studies have shown that unmet needs are negatively correlated with quality of life. They are also important predictors of psychological distress (9, 10). Unmet information and psychological needs may reduce treatment adherence. Consequently, treatment efficacy and long-term survival are negatively affected (11). For melanoma patients, special difficulties occur due to the changes in the disease condition and the infrequency of the disease. These difficulties involve image distress, social isolation and a strong fear of recurrence. Therefore, the adverse effects of unsatisfied needs are more hidden and extensive (12). Thus, the unsatisfied needs should be systematically evaluated and managed. This is an essential part of the biopsychosocial and comprehensive medical care approach.

Although unmet needs have gained increasing attention in tumor supportive care, significant research gaps remain for melanoma patients. Compared with breast and thyroid cancers (13, 14), evidence regarding the unmet needs of Chinese patients with melanoma remains limited. Most of the existing literature focuses on European and American populations. However, Asian populations differ significantly in terms of disease subtypes, cultural backgrounds, and healthcare systems. Therefore, western findings may not be directly applicable to Chinese patients. Second, methodological limitations also remain. Most studies used cross-sectional questionnaire designs (15). These can describe the distribution of unmet needs but cannot reveal the underlying reasons. Quantitative and qualitative approaches have rarely been integrated. Thus, the results are mainly described as phenomena. The underlying causes of unmet needs are difficult to investigate. Furthermore, a comprehensive theoretical model is not yet available. Unmet needs are a multi-faceted and multi-level issue that includes individual psychology, family assistance, medical facilities, and social culture. Consequently, the research outcomes are usually incomplete. It is also hard to transform these findings into effective interventions.

To solve these problems, the Social Ecological Model (SEM) (16) was applied for the qualitative analysis. SEM indicates the health-related effects at the microsystem, mesosystem and macrosystem levels and has been widely used in health promotion and disease intervention research (17–19). In melanoma patients, unmet needs may arise from personal anxiety, family and community aid, medical service accessibility and other socio-cultural aspects. Hence, SEM provides a comprehensive method to study these multiple level influences.

A convergent mixed-methods design was used to investigate the unmet needs of Chinese melanoma patients and to determine the related influencing factors. The quantitative part evaluated the unmet needs and their associated factors. The qualitative part investigated the patients’ perspectives and multiple level contextual factors by applying the Social Ecological Model. Finally, the results were combined to gain a thorough insight into the unmet needs and to guide the provision of patient-oriented, multi-level supportive care.

## 2 Methods

### 2.1 Design

This study used a convergent mixed-methods design and followed the standards of Good Reporting Practices for Mixed-Methods Studies (GRAMMS) (20).

### 2.2 Quantitative research

#### 2.2.1 Participants

Convenience sampling was used in this study. Melanoma patients were recruited from the Department of Bone and Soft Tissue Surgery at a Class III Grade A cancer Hospital in Guangxi. The recruitment period was from June 2024 to November 2025. The inclusion criteria were as follows: (1) diagnosis of melanoma; (2) awareness of their own condition; (3) age ≥ 18 years; (4) primary school education or above, with reading comprehension; and (5) informed consent and voluntary participation. The exclusion criteria were as follows: (1) other malignant tumors or organ dysfunction and (2) severe mental illness.

#### 2.2.2 Sample size calculation

Based on the analysis of the influencing factors, the required sample size was estimated to be 5 to 10 times the number of independent variables (21). With 13 independent variables, the estimated sample size was 65–130 participants. After allowing for a 20% dropout rate, the target sample size was 82–163 participants.

#### 2.2.3 Measures

General information questionnaire: The questionnaire was developed by the research team based on a literature review and group discussion. It included gender, age, occupation, education level, type of health insurance, tumor location, tumor stage, disease duration, current treatment modality, distant metastasis, and primary sources of health information (Internet-based channels, family/friends, and healthcare providers).

Needs Evaluation Questionnaire (NEQ): The NEQ was originally developed by Tamburini et al. (22) in 2000 to assess the needs of patients with cancer. The validated Chinese version developed by Gu et al. (23) in 2021 was used in this study. It demonstrated satisfactory psychometric properties, with a Cronbach’s α of 0.878, test–retest reliability of 0.922, and content validity of 0.824. The questionnaire comprises 23 items across five dimensions: information needs (9 items), nursing needs (4 items), family needs (4 items), psychological support needs (3 items), and material needs (3 items). For each item, participants responded “Yes” or “No” to indicate whether the corresponding need had been met. “No” indicates that the need remains unmet and is scored 0, whereas “Yes” indicates that the need is met and is scored 1. The total score ranges from 0 to 23, with lower scores indicating a greater number of unmet needs. This scoring direction was consistent with that of the validated Chinese version reported by Gu et al. (23). The specific items are listed in Supplementary Table 1.

#### 2.2.4 Data collection

Eligible melanoma patients were recruited with the assistance of the head nurse or attending physician. Disease-related data were extracted from medical records, and the study purpose was explained and written informed consent was obtained before questionnaire administration. A one-on-one approach was adopted for the interviews. The items were explained to the patients. For participants unable to complete the questionnaire independently, the items were read aloud and their responses were recorded by the researchers. Completeness was checked immediately, and missing responses were completed by the participants when possible.

#### 2.2.5 Data analysis

Data were analyzed using the SPSS software (version 23.0). Frequencies and percentages were used to summarize categorical data. Continuous data were summarized as mean ± SD. Group comparisons were performed using the chi-square test. Participants were dichotomized into high- and low-unmet-needs groups based on the sample mean total NEQ score (12.8). Because NEQ total scores are integer-valued, participants with scores ≤12 were classified as having high unmet needs, whereas those with scores ≥13 were classified as having low unmet needs. Variables with *p* < 0.05 in the univariate analyses were simultaneously entered into a multivariable binary logistic regression model using the enter method and were retained in the model regardless of their statistical significance after adjustment. Multicollinearity among the included predictors was assessed using tolerance values and variance inflation factors (VIFs). Adjusted odds ratios (ORs) with 95% confidence intervals (CIs) were reported. Given the strong association observed between Internet-based health information use and the potential for sparse data bias, Firth penalized logistic regression was additionally performed in R as a sensitivity analysis. All statistical tests were two-sided, and *p* < 0.05 was considered statistically significant.

#### 2.3 Qualitative research

##### 2.3.1 Participants

An independent sample of 16 participants, none of whom were included in the quantitative survey sample, was recruited in parallel with the quantitative component using maximum variation purposive sampling. The same inclusion and exclusion criteria as those used for the quantitative component were applied. Sampling continued until data saturation was reached, which was defined as the point at which additional interviews yielded no new themes or substantive information.

##### 2.3.2 Interview outline

A semi-structured interview approach was adopted, focusing on the unmet needs of melanoma patients and their related environments. The interview outline was developed as follows. A literature review was conducted. SEM (16) was used as the framework. The research objectives, team discussions, and clinical practices were considered. Expert opinions were solicited. A draft outline was then formulated and a pre-interview was conducted with three patients at different treatment stages. Based on the results, the outline was revised, and a final version was formed. The final interview outline covered three system levels: (1) Microsystem: ① What do you understand best about your melanoma (e.g., disease, treatment, care)? ② What difficulties did you face during diagnosis, treatment, and rehabilitation? How did you cope? (2) Mesosystem: ① What support do you hope your family can provide? ② Is medical team support sufficient? What help do you expect from them? Have you received peer or community support? (3) Macrosystem: Does the public or the workplace understand melanoma? Have you ever felt misunderstood or pressured?

##### 2.3.3 Data collection

Interviews were carried out face to face with the subjects, under a semi-structured format. In order to establish trust, the usual admission procedure was explained to the patients and after they gave their informed consent, the object and details of the study were further explained. One to one interviews were conducted in a peaceful room by researchers who had been trained in qualitative methods. The patients’ non-verbal actions were observed and documented. The whole interview was recorded with the permission of the patient. Several methods were applied to guarantee the accuracy, such as repetition, clarification and questioning. Each interview lasted from 30 to 40 min.

##### 2.3.4 Data analysis

Directed content analysis was conducted using the Social Ecological Model (SEM) as an *a priori* analytical framework (24). The individual, family/community, and sociocultural levels provided the overarching domains, while specific codes, subthemes, and themes were developed from the participants’ accounts. Audio recordings were transcribed verbatim within 24 h, supplemented with field notes, and checked for accuracy. Twenty percent of the transcripts were returned to the participants for verification, and the finalized transcripts were imported into NVivo 12.0 for coding and data management. Two researchers independently coded the transcripts using NVivo. Similar codes were grouped into subthemes and broader themes within the corresponding SEM domains to facilitate analysis. Coding and thematic assignments were compared between researchers, and discrepancies were resolved through discussion and re-examination of the original transcripts and field notes until a consensus was reached. Emerging themes were repeatedly checked against the original data to ensure consistency with the participants’ accounts.

#### 2.4 Integration of quantitative and qualitative research

The data were integrated using methodological triangulation. Two researchers compared the quantitative and qualitative data for consistency and differences.

#### 2.5 Ethical consideration

This study was conducted in accordance with the Declaration of Helsinki. Ethical approval was obtained from the hospital ethics committee (approval number: KY2024043). Written informed consent was obtained from all the participants. Before data collection, the study’s purpose, anonymity, and confidentiality were explained.

## 3 Results

### 3.1 Quantitative research

#### 3.1.1 Patient characteristics and univariate analysis of melanoma

Among the 105 participants, 58 (55.2%) were women, 52 (49.5%) were aged 60–84 years, and 101 (96.2%) had stage-III or IV disease. Most participants obtained health information from healthcare professionals, whereas 41 (39.0%) used Internet-based channels. Based on the sample mean total NEQ score of 12.8, 53 (50.5%) participants with scores ≤12 were classified as having high unmet needs, whereas 52 (49.5%) with scores ≥13 were classified as having low unmet needs (Table 1). In univariate analyses, gender, occupation, education, insurance type, and Internet-based channels were significantly associated with the level of unmet needs (all *p* < 0.05) (Table 2).

**Table 1:** Ranking of unmet-need dimensions from greatest to lowest (*n* = 105).

Dimension	Raw score (M ± SD)	Transformed score (M ± SD)
Information needs	3.981 ± 1.715	0.442 ± 0.191
Psychological needs	1.571 ± 1.232	0.524 ± 0.411
Material needs	1.619 ± 0.739	0.540 ± 0.246
Nursing needs	2.581 ± 0.794	0.645 ± 0.198
Family needs	3.048 ± 1.155	0.762 ± 0.289
Total score (23 points)	12.800 ± 3.652	0.557 ± 0.159

**Table 2:** Patient characteristics and univariate analysis of malignant melanoma (*n*=105).

Item	*N (%)*	Low unmet needs, 52 (49.5%)	High unmet needs, 53 (50.4%)	*X^2^*	*p*
Gender				6.070^(1)^	0.014
Male	47 (44.8)	17 (32.7%)	30 (56.6%)		
Female	58 (55.2)	35 (67.3%)	23 (43.4%)		
Age (years)				3.677^(1)^	0.159
25 ~ 44	17 (16.2)	12 (23.1%)	5 (9.4%)		
45 ~ 59	36 (34.3)	17 (32.7%)	19 (35.8%)		
60 ~ 84	52 (49.5)	23 (44.2%)	29 (54.7%)		
Occupation				14.132^(1)^	0.003
Manual worker	14 (13.3)	8 (15.4%)	6 (11.3%)		
Farmers	55 (52.4)	18 (34.6%)	37 (69.8%)		
Institutional employee	17 (16.2)	13 (25.0%)	4 (7.5%)		
Other	19 (18.1)	13 (25.0%)	6 (11.3%)		
Education level				18.463^(1)^	<0.001
Primary school or below	37 (35.2)	12 (23.1%)	25 (47.2%)		
Middle school	35 (33.3)	14 (26.9%)	21 (39.6%)		
High school/Vocational school	21 (20.0)	15 (28.8%)	6 (11.3%)		
Junior college or above	12 (11.4)	11 (21.2%)	1 (1.9%)		
Type of health insurance				15.470^(2)^	<0.001
New rural cooperative medical insurance	69 (65.7)	25 (48.1%)	44 (83.0%)		
Urban resident insurance	10 (9.5)	6 (11.5%)	4 (7.5%)		
Employee medical insurance	26 (24.8)	21 (40.4%)	5 (9.4%)		
Tumor location				1.825^(2)^	0.610
Head and face	11 (10.5)	6 (11.5%)	5 (9.4%)		
Extremities	63 (60.0)	31 (59.6%)	32 (60.4%)		
Trunk	9 (8.6)	6 (11.5%)	3 (5.7%)		
Other	22 (21.0)	9 (17.3%)	13 (24.5%)		
Tumor stage				3.959^(2)^	0.164
Stage I-II	4 (3.8)	4 (7.7%)	0		
III	55 (52.4)	26 (50.0%)	29 (54.7%)		
IV	46 (43.8)	22 (42.3%)	24 (45.3%)		
Disease duration (years)				0.462^(1)^	0.794
<1	25 (23.8)	11 (21.2%)	14 (26.4%)		
1 ~ 3	36 (34.3)	19 (36.5%)	17 (32.1%)		
>3	44 (41.9)	22 (42.3%)	22 (41.5%)		
Current treatment modality				2.656^(2)^	0.633
Surgery	44 (41.9)	22 (42.3%)	22 (41.5%)		
Radiotherapy	6 (5.7)	3 (5.8%)	3 (5.7%)		
Chemotherapy	15 (14.3)	5 (9.6%)	10 (18.9%)		
Immunotherapy	31 (29.5)	18 (34.6%)	13 (24.5%)		
Targeted therapy	9 (8.6)	4 (7.7%)	5 (9.4%)		
Distant metastasis				1.600^(1)^	0.206
No	48 (45.7)	27 (51.9%)	21 (39.6%)		
Yes	57 (54.3)	25 (48.1%)	32 (60.4%)		
Current primary source of health information					
(1) Internet-based channels				34.568^(1)^	<0.001
No	64 (61.0)	17 (32.7%)	47 (88.7%)		
Yes	41 (39.0)	35 (67.3%)	6 (11.3%)		
(2) Family/friends channels				3.583^(1)^	0.058
No	39 (37.1)	24 (46.2%)	15 (28.3%)		
Yes	66 (62.9)	28 (53.8%)	38 (71.7%)		
(3) Healthcare provider channels				0.401^(1)^	0.527
No	25 (23.8)	11 (21.2%)	14 (26.4%)		
Yes	80 (76.2)	41 (78.8%)	39 (73.6%)		

(1) Asymptotic method was used for chi-square test; (2) Fisher’s exact test was used when expected cell frequencies were <5.

#### 3.1.2 Ranking of need dimensions

Due to the varying item numbers across the NEQ dimensions, each dimension score was calculated as the raw total divided by its item count. A lower score indicates greater unmet need. The information dimension showed the greatest level of unmet need, followed by the psychological, material, nursing, and family dimensions (Table 1). The top 5 items in the frequency of demand in the NEQ questionnaire are shown in Supplementary Table 2, four items fall under the information dimension, and one item belongs to the nursing dimension. A heatmap showing the unmet needs for each item in melanoma patients is presented in Figure 1.

**Figure 1:**
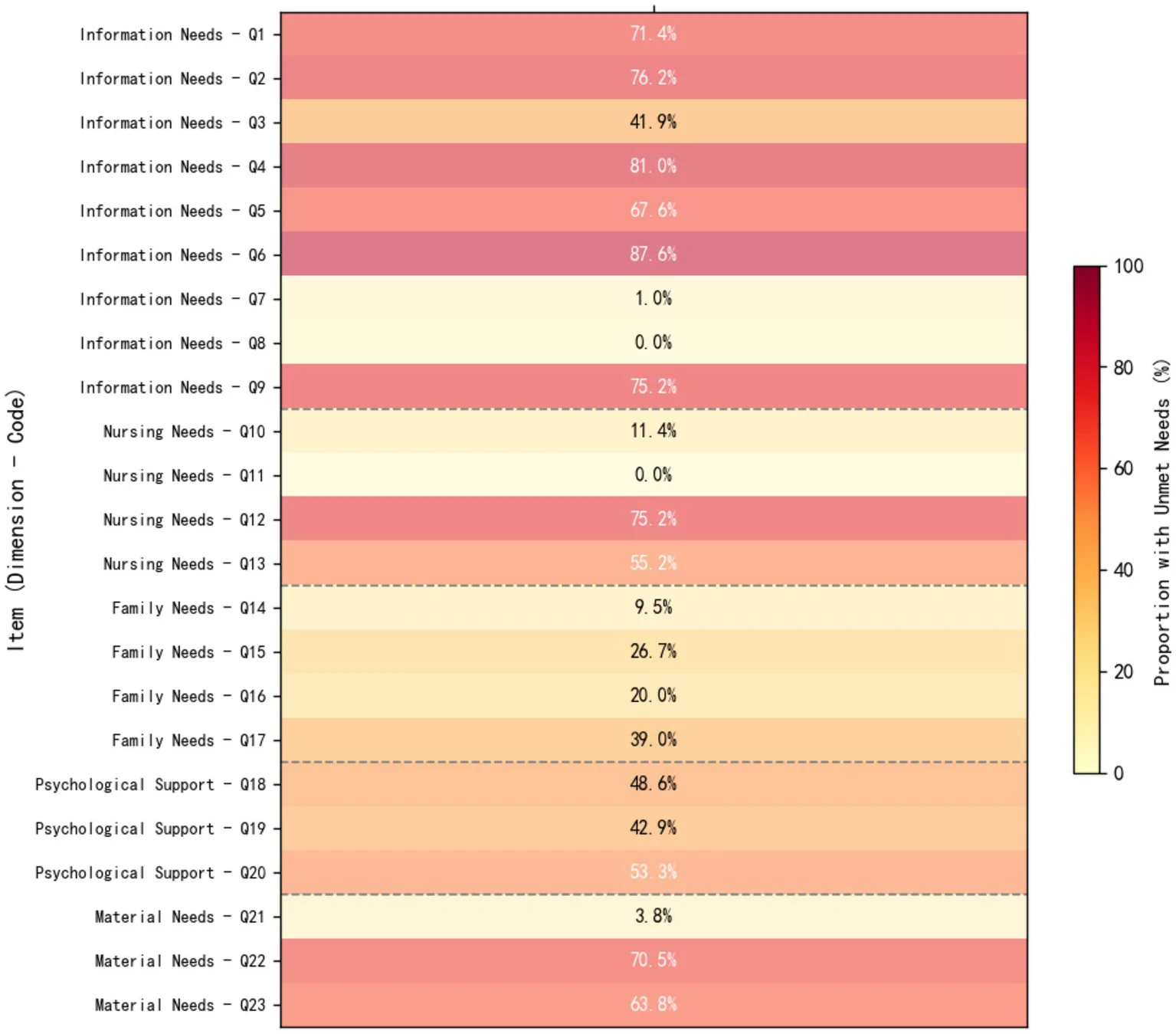
Heatmap of unmet needs by item in melanoma patients (yellow indicates lower needs; red indicates higher needs) proportion with unmet needs (%). ① Q1–Q9: information needs; ② Q10–Q13: nursing needs; ③ Q14–Q17: family needs; ④ Q18–Q20: psychological needs; ⑤ Q21–Q23: materials needs.

#### 3.1.3 Multivariable binary logistic regression analysis of unmet needs in patients with melanoma

The independent variables and their coding are presented in Supplementary Table 3. Variables with *p* < 0.05 in the univariate analyses were simultaneously entered into the multivariable binary logistic regression model. No problematic multicollinearity was identified among the included predictors, with tolerance values ranging from 0.380 to 0.911 and variance inflation factors (VIFs) ranging from 1.098 to 2.631 (Supplementary Table 4).

In the multivariable model, gender and use of Internet-based health information channels remained significantly associated with unmet need group membership. Compared with male patients, female patients had lower odds of belonging to the high-unmet-needs group (*OR* = 0.257, *95%CI*: 0.080–0.830, *p* = 0.023). Similarly, patients who used Internet-based health information channels had lower odds of belonging to the high-unmet-needs group than those who did not (*OR* = 0.075, 95%*CI*:0.022–0.257, *p* < 0.001). Occupation, education level, and type of health insurance were not significantly associated with unmet needs group membership after adjustment (overall *p* = 0.161, 0.611, and 0.310, respectively) (Table 3). Given the strong association between Internet-based health information use and unmet needs, a Firth penalized logistic regression was additionally performed as a sensitivity analysis. The association remained statistically significant and in the same direction (OR = 0.108, 95%*CI*:0.033–0.309, *p* < 0.001) (Supplementary Table 5).

**Table 3:** Multivariable binary logistic regression analysis of unmet needs among patients with melanoma (*n* = 105).

Variables	*B*	*S.E.*	*Wald X^2^*	*p*	*OR*	*95%CI*
Constant	0.888	0.763	1.352	0.245	2.429	—
Gender						
Female	−1.359	0.598	5.162	0.023	0.257	0.080–0.830
Occupation		5.157		0.161		
Farmer	1.780	0.819	4.730	0.030	5.932	1.192–29.514
Institutional employee	0.677	1.153	0.345	0.557	1.968	0.205–18.877
Other	1.262	1.144	1.217	0.270	3.532	0.375–33.255
Education level		1.819		0.611		
Middle school	0.284	0.648	0.192	0.661	1.328	0.373–4.726
High/vocational school	−0.176	0.886	0.039	0.843	0.839	0.148–4.763
Junior college or above	−1.488	1.371	1.178	0.278	0.226	0.015–3.317
Type of health insurance		2.345		0.310		
Urban resident insurance	−1.240	0.964	1.655	0.198	0.289	0.044–1.915
Employee medical insurance	−1.164	0.980	1.410	0.235	0.312	0.046–2.132
Internet-based health-information channels						
Yes	−2.585	0.626	17.065	<0.001	0.075	0.022–0.257

B, regression coefficient; S.E., standard error; OR, odds ratio; CI, confidence interval. Male sex, manual worker, primary school or below, new rural cooperative medical insurance, and non-use of Internet-based health-information channels were the reference categories. The low-unmet-needs group was coded as 0 and the high-unmet-needs group as 1. Model performance: Omnibus *χ*^2^ (10) = 57.604, *p* < 0.001; Nagelkerke *R*^2^ = 0.563; Hosmer–Lemeshow *χ*^2^ (8) = 5.765, *p* = 0.674; overall classification accuracy = 83.8%.

### 3.2 Qualitative research

Sixteen melanoma patients completed valid interviews and were coded P1 to P16. The age range of these patients was 44–71 years (Supplementary Table 6). Based on SEM, themes were categorized into three levels: microsystem, mesosystem, and macrosystem. Nine themes were identified in this study. Notable patient quotations are presented in Supplementary Table 7.

#### 3.2.1 Individual-level (microsystem)

##### 3.2.1.1 Disease perception and health education needs

- *Lack of knowledge and awareness regarding early recognition*. A total of 75% (12/16) of patients directly reported having no knowledge of melanoma before diagnosis, often mistaking the lesions for ‘dark lines,’ ‘corns,’ or ‘common moles,’ and even resorting to folk remedies, leading to delayed diagnosis.
- *Misperceptions regarding disease severity*. A subset of patients demonstrated an unclear understanding of the malignant nature of melanoma, likening it to chronic conditions such as diabetes mellitus, thereby underestimating its invasive potential.
- *Passive knowledge acquisition and the need for information clarification*. Patients obtained incomplete information from non-specialist sources such as their children and internet materials, and they had a need for clear and detailed advice on diet and recovery.

##### 3.2.1.2 Complex and multidimensional emotional distress

- *Disease uncertainty and fear of prognosis*. From anxiety and insomnia before diagnosis to hypervigilance toward physical signs after diagnosis, as well as anxiety and distress when facing disease progression, uncertainty about the future constituted the core psychological burden.
- *Fear and distress regarding treatment side effects*. Five patients (31.3%) reported physical and psychological suffering caused by symptoms such as pain, walking impairment, edema, ascites, and poor sleep quality after treatment, with some even experiencing thoughts of giving up treatment.
- *Stigma and social image disruption*. After operation, restricted walking or wound deterioration caused patients to be afraid of being criticized, laughed at or rejected by others. Therefore, they avoided contact with others and felt lonely and detached from the world.

##### 3.2.1.3 Cognitive restructuring and life adjustment

- *Seeking a sense of control and active coping*. Some individuals altered their opinions and established fixed habits. They tried to improve their immunity through nutritional supplements and acupuncture, obtained spiritual assistance, or were encouraged by their sense of family obligation.

#### 3.2.2 Family /community-level (mesosystem)

##### 3.2.2.1 The double-edged sword effect of family support

- *Coexistence of feeling loved and burdened*. Attentive family care provided patients with emotional comfort. However, participants reported that frequent inquiries from family members reinforced their perceived dependence and increased their psychological burden. Meanwhile, the financial stress caused by the illness also brought a profound sense of guilt.

##### 3.2.2.2 Peer support

- *Sharing of information and experiences*. Exchanging insights with peers provided patients with direct experiential information about the disease and its management. This not only reduced anxiety stemming from uncertainty but also facilitated the restoration of treatment-related self-confidence among patients.

##### 3.2.2.3 Trust and trauma in clinician-patient communication

- *Unintended harm in information delivery*. Even ordinary comments from medical staff or private talks among family members without the patient present may greatly increase the patient’s suspicion and anxiety about their health condition and treatment.
- *Emotional support from healthcare providers.* Healthcare providers’ reassurance and encouragement provided significant emotional support during treatment. Participants mentioned that the reassurance and encouragement from physicians were important sources of emotional comfort during treatment.
- *Desire for diversified health education*. Besides receiving passive information, patients demanded that hospitals use multichannel teaching methods, such as telephone consultations, messages on WeChat, printed documents, and combined online and offline group meetings. Their purpose was not only to acquire knowledge but also to find true friends and gain a stronger sense of belonging.

#### 3.2.3 Social/cultural-level (macrosystem)

##### 3.2.3.1 Barriers to healthcare access and continuity of care

- *Misdiagnosis and missed diagnosis at the primary care level*. Patients often felt unhappy about the incorrect first diagnosis in the neighbouring hospitals. At the community health centres or general hospitals, the strange skin marks were usually confused with ordinary diseases like viral infections, corns or common moles. They thought that these mistaken diagnoses resulted in unnecessary delays and postponed the proper treatment.
- *Difficulty in accessing specialist resources*. Patients often encounter problems in locating melanoma specialists from the local health services. The shortage of local experts forces them to seek specialized medical treatments in other areas, particularly by using the Internet and various networks to find appropriate treatment centers. The geographical and information obstacles result in a delay in obtaining a definite diagnosis and proper treatment.
- *Insufficient doctor-patient communication in treatment planning*. Patients had an inadequate understanding of core information, such as surgical complications and subsequent treatment regimens, reflecting a lack of fully informed communication during the clinical decision-making process.

##### 3.2.3.2 Heavy economic burden

- *The double blow of illness-induced poverty and income disruption*. High treatment costs and reduced household income created substantial financial pressure. Some patients hesitated to undergo effective treatments, such as chemotherapy, due to concerns about the economic burden.

##### 3.2.3.3 The shackles of sociocultural beliefs and their breakthrough

- *Disease stigmatization*. Participants described cancer-related stigma and misconceptions about contagion as contributing to social isolation.
- *Digital divide and health literacy disparities*. Older adults or less educated patients, who are not proficient in using the Internet, were unable to access effective information and thus rely more on traditional forms of health education (e.g., telephone calls, videos, brochures). Moreover, patients in rural areas exhibit even more pronounced deficits in disease knowledge.

### 3.3 Joint display of quantitative and qualitative findings

The results of this integration are presented in Table 4.

**Table 4:** Joint display.

System level	Qualitative theme and key finding	Quantitative finding	Relationship	Integrated interpretation
Individual-level (microsystem)	1. Disease perception and health education needs: Limited disease awareness, misconceptions, and reliance on fragmented information.	Information needs ranked highest; 87.6% required understandable disease information, 81.0% required treatment information, and 76.2% required information about disease progression.	Convergence	Knowledge gaps and fragmented information sources were consistent with patients’ substantial need for clear and authoritative information about melanoma, treatment, and prognosis.
	2. Complex and multidimensional emotional distress: Distress arising from disease uncertainty, treatment-related effects, altered body image, and perceived stigma.	Psychological needs ranked second; 48.6% required psychological support. Female sex was associated with lower odds of high unmet needs (*OR* = 0.257, *95% CI*: 0.080–0.830, *p* = 0.023).	Complementarity	The interviews identified the major sources of these psychological needs. However, the lower odds of high unmet needs among women were not fully explained by qualitative findings.
	3. Cognitive restructuring and life adjustment: Patients used cognitive reframing, lifestyle adjustments, spiritual support, and family responsibility to regain a sense of control.	No directly corresponding indicator was included in the NEQ.	Expansion	The interviews identified active coping and meaning-making processes that were not captured in the quantitative assessment.
Family/community-level (mesosystem)	4. The double-edged nature of family support: Family care provided comfort but also reinforced the patient’s role and generated feelings of guilt and burden.	Family needs ranked the lowest; with 9.5% of respondents reporting a need for greater reassurance from family members.	Complementarity	The qualitative findings clarified how low reported family needs could coexist with family-related distress: family care provided support, while concerns about dependence and burdening family members remained.
	5. Peer support: Peer communication provides experiential information and emotional validation.	A total of 53.3% of the participants wanted to communicate with other patients.	Convergence	Both findings indicate that patients value peer communication as a source of shared experience and emotional support.
	6. Trust and trauma in clinician–patient communication: Communication influences trust, anxiety, and willingness to seek information, while patients prefer multiple health-education channels.	Information needs ranked highest; 75.2% required greater attention from nurses, and 75.2% required greater reassurance from clinicians.	Complementarity	The qualitative findings showed that unmet information and care needs involved not only the amount of information provided but also the communication quality, emotional sensitivity, timing, and mode of delivery.
Social/cultural-level (macrosystem)	7. Barriers to healthcare access and continuity of care: Misdiagnosis, specialist shortages, geographical barriers, and insufficient treatment communication impeded access to appropriate care.	Occupation was associated with unmet-needs group membership in the univariate analysis (*p* = 0.003), but the association was not significant after multivariable adjustment (overall *p* = 0.161).	Complementarity	The attenuation of the association with occupation after adjustment suggests that occupational differences in unmet needs may reflect broader contextual factors rather than occupation. The qualitative findings complemented this result by identifying specific barriers to healthcare access.
	8. Heavy economic burden: High treatment costs and reduced household income threaten treatment affordability and continuity.	Material needs ranked third; 63.8% required financial assistance, and 70.5% required information about health insurance or reimbursement.	Convergence	Both findings indicate that direct treatment costs and illness-related income loss contribute to patients’ financial and insurance-related needs.
	9a. Digital divide and health literacy disparities: Limited digital skills restrict access to and evaluation of reliable online health information.	The use of Internet-based channels was associated with lower odds of high unmet needs (*OR* = 0.075, *95% CI*: 0.022–0.257, *p* < 0.001).	Complementarity	The interviews suggested that Internet access could facilitate health information acquisition, but its potential benefits depended on patients’ digital skills and ability to evaluate online information.
	9b. Disease stigmatization: Fear of cancer and misconceptions about contagion contribute to social avoidance and isolation.	The NEQ does not directly measure cancer-related stigma.	Expansion	The interviews identified cancer-related stigma as an additional source of social and psychological distress that was not captured by quantitative assessment.

Convergence indicates that the quantitative and qualitative findings supported the same conclusions. Complementarity indicates that one set of findings provides contextual or explanatory insights into the other. Expansion indicates that the qualitative findings identified experiences or processes that were not measured in the quantitative component. OR, odds ratio; NEQ, Needs Evaluation Questionnaire.

## 4 Discussion

This study explored unmet needs and their influencing factors in melanoma patients using a convergent mixed-methods design. All patients reported at least one unmet need, with information needs being the most prominent. Based on SEM, nine themes were organized into microsystem, mesosystem, and macrosystem perspectives. Microsystem-level factors were prominent, particularly limited recognition of early symptoms and poor disease awareness. These factors may contribute to delayed recognition and healthcare-seeking, potentially increasing the likelihood of diagnosis at a more advanced stage.

According to Fu et al. (15), the main unmet demands in this research were information needs. The quantitative results indicated that the frequently mentioned demands were information concerning the disease (87.6%), treatment (81.0%), disease progression (76.2%) and the attention from the clinicians (75.2%). These results are also consistent with a study conducted in Poland using the same questionnaire (25). In contrast, Thompson et al. (26) reported that psychological and emotional needs were more prominent. This difference may be due to variations in diseases and medical systems. Australia has one of the highest melanoma incidence rates in the world (1), but it also provides extensive public education and thorough examination (27). Consequently, melanoma is more likely to be detected at an earlier stage, when surgical treatment may be associated with more favorable outcomes. In contrast, melanoma is rarely found in the Asian population, with a much lower incidence rate than in Europe and America (2). The relatively low incidence of melanoma in China may contribute to limited public awareness. Moreover, knowledge of skin cancer in China is insufficient. Public education regarding melanoma remains insufficient, and melanoma-related health education is limited in educational settings (28). Qualitative findings showed that 75% of participants had no knowledge of melanoma before diagnosis, highlighting substantial gaps in disease awareness. Separately, some participants underestimated the severity of melanoma, which may have contributed to delayed healthcare-seeking. There are two major macro-system factors that prevent patients from obtaining more information: poor doctor-patient communication and the digital gap. Based on these results, several suggestions have been proposed. Health education materials suitable for Chinese culture should be developed. Public education should be strengthened to raise awareness of early stage melanoma. Training for general practitioners and dermatologists should be improved. Specialized diagnostic and treatment departments and effective cooperation systems should be established. A formal online health information website should be established to provide personalized advice, manuals, and short videos. These measures offer comprehensive assistance to patients (29).

Psychological distress is common in melanoma patients, including anxiety, depression, and fear of recurrence (30). This distress affects mental health, treatment compliance, and outcomes (31), consistent with the present findings. Psychological needs ranked second only to information needs. Qualitative data showed that treatment side effects aggravated distress at the microsystem level. This is supported by Reitmajer et al. (32), who linked persistent adverse events to psychosocial distress. Unmet information needs have also been associated with isolation, uncertainty, and psychological distress (33). At the mesosystem and macrosystem levels, communication barriers and disease stigma increased anxiety and helplessness, and patients’ psychological states evolved with disease progression. Initial negative emotions were gradually replaced by acceptance and positive coping, aligning with post-traumatic growth (34). The patients said that they felt fewer negative emotions with the help of strong social support and encouragement from doctors. A kind and supportive relationship makes the care environment more attractive and encouraging (35). Based on these findings, several recommendations can be made. Medical workers should not only promote medical information but also consider the mental health. An adjustable psychological assessment system should be set up to offer personalized treatment. In all, these actions will influence the patient’s short-term and medium-term social systems, working together with superior policies to decrease the psychological difficulties experienced by melanoma patients.

Regarding economic burden, 63.8% of patients sought financial aid, and 70.5% requested information about insurance. These quantitative findings were supported by qualitative reports of substantial financial burden. This result is opposite to the study conducted in Germany (32), where only 8% referred to financial restrictions. This difference might be caused by differences in patients’ conditions and health care systems. Most of the participants in that study were unemployed people close to retirement age, whereas in our study, more than half of the patients were rural inhabitants who were insured. In China, cancer patients often experience financial difficulties because although the main medical expenses are covered, out-of-pocket costs are still high, and the procedures for reimbursement of out-of-town treatment are complicated (36). These factors substantially affect treatment decisions in low-income groups. Economic risks should be screened during the initial diagnosis. Individualized follow-up plans should be developed. Patients should be helped to understand insurance reimbursement and assisted with applications for special disease-related benefits. These measures would help relieve the economic pressure.

In the multivariable model, sex and use of Internet-based health information channels remained significantly associated with unmet needs group membership. Female patients had lower odds of high unmet needs than male patients, and patients who used Internet-based health information channels had lower odds of high unmet needs than those who did not. Compared with conventional approaches, the Internet facilitates active search, continuous access, and multiple sources of verification. These features may facilitate access to information across diagnosis, treatment, and rehabilitation and may reduce uncertainty (37). Female patients had lower odds of high unmet needs than male patients, consistent with the findings of Osowiecka et al. (25). The interviews suggested that some middle-aged male patients experienced substantial treatment-related and financial burdens. Other studies have obtained opposite results. Lou et al. (38) reported greater supportive care needs among female cancer survivors in China, Zhang et al. (39) also reported greater supportive care needs among women with colorectal cancer. These findings show that the supportive care must consider the particular needs, psychological and socioeconomic aspects of male patients. Special psychological help and personalized re-employment plans should be provided for these patients.

Occupation was associated with unmet needs group membership in the univariate analysis but was not significant as an overall factor after multivariable adjustment. Nevertheless, qualitative interviews highlighted limited access to specialized care and low health literacy as important contextual barriers, consistent with previous evidence linking socioeconomic disadvantage and limited healthcare access to unmet needs among patients with advanced cancer (40). Therefore strengthening primary-level follow-up, health education, referral pathways, and access to specialist support may help reduce healthcare access disparities.

### 4.1 Limitations

This study had several limitations. First, patients were recruited from a single tertiary care hospital in the Guangxi Zhuang Autonomous Region using a non-probability sampling strategy. Although several demographic characteristics were broadly comparable to those reported in previous Chinese melanoma cohorts, our sample contained a higher proportion of patients with advanced-stage disease (41, 42). Thus, the sample may disproportionately represent patients with more advanced disease, and the generalizability of the findings to the broader melanoma population should be interpreted with caution. Second, the cross-sectional design precludes causal inference and does not capture changes in unmet needs across the disease trajectory. Third, the relatively small quantitative sample and limited number of outcome events may have reduced the stability and precision of multivariable estimates. Fourth, psychological distress and quality of life were not assessed using standardized instruments. Therefore, related interpretations were based solely on the NEQ psychological support dimension and qualitative interview data. Finally, the dichotomization of NEQ scores was based on the sample mean rather than a validated clinical cutoff, which may limit the generalizability of this classification.

### 4.2 Implications for clinical practice and health policy

The integrated findings highlight the need for a more needs-oriented model of melanoma care. Clinically, unmet needs should be assessed routinely and addressed through individualized information provision, supportive communication, and timely referral to psychological, financial, or other supportive services. At the system level, strengthening primary care recognition of melanoma, improving referral pathways and access to specialist expertise, and expanding reliable digital and telehealth resources may help reduce barriers related to information, geography, and continuity of care.

## 5 Conclusion

This mixed-methods study identified information needs as a prominent unmet need among patients with melanoma. Female sex and the use of Internet-based health information channels were associated with lower odds of high unmet needs. Qualitative findings further contextualized the quantitative results by identifying interacting influences across individual, family/community, and sociocultural levels, including limited disease awareness, emotional concerns, family and peer support, healthcare access barriers, financial burden, and disparities in digital health literacy. These findings highlight the need for coordinated, needs-oriented, and supportive care for patients with melanoma.

## Data Availability

The raw data supporting the conclusions of this article will be made available by the authors, without undue reservation.
