# Supplementary material for: Unmet needs and associated factors among patients with melanoma: a mixed-methods study
Source: Front Public Health. 2026 Sep 28;14:1956448. doi: 10.3389/fpubh.2026.1956448 (PMC13655005; doi:10.3389/fpubh.2026.1956448)
Supplement: Supplementary file 1 [file Table_1.docx]

**Supplemental Table 1 Tthe Needs Evaluation Questionnaire (NEQ)**

| **Dimension** | **No.** | **Item** |
| --- | --- | --- |
| Information needs | Q1 | I need more information about my diagnosis. |
|  | Q2 | I need more information about my future condition. |
|  | Q3 | I need more information about the exams I am undergoing. |
|  | Q4 | I need more explanations of treatments. |
|  | Q5 | I need to be more involved in the therapeutic choices. |
|  | Q6 | I need clinicians and nurses to give me more comprehensible information. |
|  | Q7 | I need clinicians to be more sincere with me. |
|  | Q8 | I need to have a better dialogue with clinicians. |
|  | Q9 | I need to be more reassured by the clinicians. |
| Nursing needs | Q10 | I need more help with eating, dressing, and going to the bathroom. |
|  | Q11 | I need better respect for my intimacy. |
|  | Q12 | I need better attention from nurses. |
|  | Q13 | I need my symptoms (pain, nausea, insomnia, etc.) to be better controlled. |
| Family needs | Q14 | I need to be more reassured by my relatives. |
|  | Q15 | I need to feel more useful within my family. |
|  | Q16 | I need to feel less abandoned. |
|  | Q17 | I need to receive less commiseration from other people. |
| Psychological support | Q18 | I need to speak with a psychologist. |
|  | Q19 | I need to speak with a spiritual advisor. |
|  | Q20 | I need to speak with people who have this same experience. |
| Material needs | Q21 | I need better services from the hospital (bathrooms, meals, cleaning). |
|  | Q22 | I need to have more economic insurance information (tickets, invalidity, etc.) in relation to my illness |
|  | Q23 | I need economic help. |

**Supplemental Table 2 Top 5 items ranked by need frequency in the NEQ questionnaire (*n*=105)**

| **Rank** | **Item** | **Dimension** | ***N*(%)** |
| --- | --- | --- | --- |
| 1 | Q6: I need clinicians and nurses to give me more comprehensible information. | Information needs | 92(87.6%) |
| 2 | Q4: I need more explanations of treatments. | Information needs | 85(81.0%) |
| 3 | Q2: I need more information about my future condition. | Information needs | 80(76.2%) |
| 4 | Q9: I need to be more reassured by the clinicians. | Information needs | 79(75.2%) |
| 5 | Q12: I need better attention from nurses. | Nursing needs | 79(75.2%) |

**Supplemental Table 3 Coding of variables included in the multivariable logistic regression(*n*=105)**

| **Variable** | **Category** | **Coding** |
| --- | --- | --- |
| **Unmet-needs group** | Low unmet needs | 0 |
|  | High unmet needs | 1 |
| **Gender** | Male | 0 |
|  | Female | 1 |
| **Occupation** | Manual worker | Reference:(0, 0, 0) |
|  | Farmer | (1, 0, 0) |
|  | Institutional employee | (0, 1, 0) |
|  | Other | (0, 0, 1) |
| **Education level** | Primary school or below | Reference: (0, 0, 0) |
|  | Middle school | (1, 0, 0) |
|  | High school/vocational school | (0, 1, 0) |
|  | Junior college or above | (0, 0, 1) |
| **Type of health insurance** | New rural cooperative medical insurance | Reference: (0, 0) |
|  | Urban resident insurance | (1, 0) |
|  | Employee medical insurance | (0, 1) |
| **Internet-based channels** | No | 0 |
|  | Yes | 1 |

**Supplemental Table 4 Multicollinearity diagnostics for variables included in the multivariable logistic regression model(*n*=105)**

| **Predictor** | **Tolerance** | **VIF** |
| --- | --- | --- |
| **Gender** | 0.911 | 1.098 |
| **Internet-based health-information channels** | 0.751 | 1.332 |
| **Occupation** |  |  |
| Farmer | 0.387 | 2.584 |
| Institutional employee | 0.421 | 2.374 |
| Other | 0.380 | 2.631 |
| **Education level** |  |  |
| Middle school | 0.726 | 1.378 |
| High school/vocational school | 0.525 | 1.904 |
| Junior college or above | 0.538 | 1.859 |
| **Type of health insurance** |  |  |
| Urban resident insurance | 0.790 | 1.266 |
| Employee medical insurance | 0.481 | 2.080 |

Note: VIF, variance inflation factor. Reference categories were male for gender, manual worker for occupation, primary school or below for education level, new rural cooperative medical insurance for insurance type, and non-use for Internet-based health-information channels.

**Supplemental Table 5 Sensitivity analysis using Firth penalized logistic regression (n=105)**

| **Predictor** | **Conventional logistic OR (95%CI)** | ***P*** | **Firth logistic OR**  **(95% CI)** | ***P*** |
| --- | --- | --- | --- | --- |
| **Gender** |  |  |  |  |
| Female | 0.257 (0.080–0.830) | 0.023 | 0.310 (0.100–0.870) | 0.026 |
| **Occupation** |  |  |  |  |
| Farmer | 5.932 (1.192–29.514) | 0.030 | 4.654 (1.080–21.621) | 0.039 |
| Institutional employee | 1.968 (0.205–18.877) | 0.557 | 1.812 (0.239–16.524) | 0.571 |
| Other | 3.532 (0.375–33.255) | 0.270 | 2.846 (0.383–24.478) | 0.311 |
| **Education level** |  |  |  |  |
| Middle school | 1.328 (0.373–4.726) | 0.661 | 1.238 (0.385–4.149) | 0.720 |
| High/vocational school | 0.839 (0.148–4.763) | 0.843 | 0.850(0.174–4.481) | 0.842 |
| Junior college | 0.226 (0.015–3.317) | 0.278 | 0.337 (0.024–2.932) | 0.334 |
| **Type of health insurance** |  |  |  |  |
| Urban resident | 0.289 (0.044–1.915) | 0.198 | 0.347 (0.059–2.046) | 0.237 |
| Employee medical | 0.312 (0.046–2.132) | 0.235 | 0.377 (0.062–2.132) | 0.270 |
| **Internet-based health-information channels** |  |  |  |  |
| Yes | 0.075 (0.022–0.257) | xiao小于  <0.001 | 0.108 (0.033–0.309) | <0.001 |

Note: OR, odds ratio; CI, confidence interval. The high-unmet-needs group was modeled as the outcome event. Reference categories were male sex, manual worker, primary school or below, new rural cooperative medical insurance, and non-use of Internet-based health-information channels.

**Supplemental Table 6 General Information Form for Interviews with Melanoma Patients (*n* = 16)**

| **No.** | **Gender** | **Age** | **Occupation** | **Education level** | **Location of melanoma** | **Duration of illness**  **(months)** | **Tumor staging** | **Complication** | **Treatment status** | **Average monthly household income**  **(yuan)** |
| --- | --- | --- | --- | --- | --- | --- | --- | --- | --- | --- |
| P01 | male | 56 | farmer | ② | Right thumb | 48 | IIb | Hypertension | Radiotherapy and Immunotherapy | <3000 |
| P02 | female | 78 | retiree | ③ | Right foot | 60 | IIIC | N | Before surgery | 5001-8000 |
| P03 | male | 57 | manual worker | ① | Left plantar | 72 | Ⅲb | N | Before surgery | 5001-8000 |
| P04 | female | 69 | retiree | ④ | Left eyelid | 84 | Ⅱb | N | Chemotherapy and immunotherapy | ＞8000 |
| P05 | female | 65 | retiree | ② | Left plantar | 36 | Ⅲb | Diabetes | Targeted Therapy | ＞8000 |
| P06 | male | 53 | manual worker | ② | Right plantar | 4 | Ⅳa | Bone metastasis | Immunotherapy | 3001～5000 |
| P07 | male | 44 | driver | ① | Right plantar | 120 | Ⅲa | N | Before surgery | <3000 |
| P08 | male | 62 | retiree | ② | Left groin | 36 | Ⅲa | Hypertension | Chemotherapy and immunotherapy | 5001-8000 |
| P09 | female | 56 | retiree | ① | Left plantar | 8 | Ⅲb | Diabetes | Chemotherapy and immunotherapy | 3001～5000 |
| P10 | female | 71 | retiree | ② | Left heel | 240 | Ⅱa | Hypertension and Diabetes | Surgery | 3001～5000 |
| P11 | male | 45 | manual worker | ① | Right axilla | 10 | Ⅳa | Peritoneal metastasis | Targeted Therapy | <3000 |
| P12 | male | 60 | farmer | ② | Left neck | 12 | Ⅲa | Diabetes | Before surgery | ＞8000 |
| P13 | male | 55 | manual worker | ① | Left heel | 24 | Ⅱb | N | Surgery | 5001-8000 |
| P14 | female | 57 | farmer | ① | Left plantar | 36 | Ⅲa | N | Radiotherapy and Immunotherapy | ＞8000 |
| P15 | male | 59 | teacher | ④ | Right plantar | 120 | Ⅲc | N | Chemotherapy and immunotherapy | 5001-8000 |
| P16 | female | 62 | retiree | ① | Urethra | 1 | Ⅲc | Hypertension and Diabetes | Before surgery | 3001～5000 |

Note: Education level: ①Primary school; ②Middle school; ③High school/Vocational school; ④Junior college;

**Supplemental Table 7 Indicative quotations**

| Level | Theme | Subtopic | No. | Representative participants quotations |
| --- | --- | --- | --- | --- |
| Individual-level (microsystem) | Disease Perception and Health Education Needs. | Lack of knowledge and awareness regarding early recognition. | P02 | When it was first discovered, I thought it was a corn. I applied herbal remedies at home for a while, but the wound became ulcerated instead. |
|  |  |  | P04 | Three years ago, I noticed a black line appearing at the corner of my eye, and I always thought it was a normal phenomenon. |
|  |  | Misperceptions regarding disease severity. | P08 | After returning home from surgery, I didn't pay special attention to postoperative recovery matters and just maintained my original living habits. |
|  |  |  | P12 | My child told me it was a tumor, but I thought this disease was probably similar to diabetes and didn't affect my daily life. |
|  |  | Passive knowledge acquisition and the need for information clarification. | P05 | Nowadays, I rarely eat foods considered to be 'hair-trigger foods' (fatty, spicy, or allergenic foods in traditional Chinese medicine). I also take some Chinese herbs to replenish qi and blood to boost my immunity every day. It would be even better if there was a scientific diet plan. |
|  |  |  | P09 | All the knowledge about diet was told to me by my child—eating eggs, milk, and two kinds of fruits. I don't know if this is correct or not. |
|  | Complex and multidimensional emotional distress. | Disease uncertainty and fear of prognosis. | P15 | A few years ago, I suddenly found out that nodules had appeared in my lungs and liver, which made me suspect that this was a sign that the disease was progressing too quickly. I always felt a sense of unease inside. |
|  |  |  | P05 | The heaviest psychological burden was before diagnosis, watching it grow little by little.Panic and anxiety kept me awake at night. |
|  |  |  | P01 | Now that tests have found metastasis, I am still quite anxious. |
|  |  | Fear and distress regarding treatment side effects. | P04 | After the fourth session of immunotherapy, my limbs became severely swollen, and I felt weak all over. |
|  |  |  | P11 | After taking the targeted therapy drug, I experienced recurrent fever and skin allergies. |
|  |  |  | P14 | The severe pain during radiotherapy made me want to give up (eyes moist, voice choked). I often took painkillers, but the repeated episodes truly exhausted me both physically and mentally. |
|  |  | Stigma and Social Image Disruption. | P02 | Sometime after the surgery, my wound ulcerated and discharged pus. When people saw it, they didn't dare come near me. That really hurt inside. |
|  |  |  | P16 | I'm scared to go out in my wheelchair. I always think people are wondering what's wrong with me. Over time, I've really become withdrawn. |
|  | Cognitive restructuring and life adjustment | Seeking a sense of control and active coping. | P03 | Later, I came to terms with it myself and started sleeping regularly every day. |
|  |  |  | P05 | My child told me that most people nowadays live with tumors. Hearing that made me feel quite relieved and made me trust current medical technology more. At the same time, I want to use spiritual strength to overcome it. |
|  |  |  | P11 | My only wish is to see my children go further in life. That is also my main spiritual pillar. |
| Family /Community-Level (Mesosystem) | The double-edged sword effect of family support. | Coexistence of feeling loved and feeling burdened. | P07 | If the treatment costs 200,000 to 300,000 yuan, I wouldn't be able to afford it. I feel like I'm becoming a financial burden on my family. |
|  |  |  | P15 | My family took very good care of me during chemotherapy. I felt very warm inside. |
|  |  |  | P16 | My family constantly ask about my body and test results, which magnifies my identity as a patient. I wish I could occasionally set aside the illness and talk about our children or movies, and just be a normal husband and father again. |
|  | Peer support | Sharing of information and experiences. | P09 | After coming to the cancer hospital, I saw that every room was filled with cancer patients—I wasn't the only one. After talking with them, I learned that people can live quite a long time, and I regained my confidence. |
|  |  |  | P11 | I like chatting with others. Those fellow patients taught me a lot about this disease. Gradually, I no longer felt the fear of the unknown about it. |
|  | Trust and trauma in clinician-patient communication | Unintended harm in information delivery. | P10 | Last time during the doctor's rounds, I heard the attending physician say that this was a 'bad thing.' I was terrified at that moment. Whenever I see doctors calling my family aside for private conversations, I become even more worried about my condition. |
|  |  | The spiritual comfort role of healthcare providers. | P14 | The doctor really helped me a lot. Whenever I was suffering so much from the pain that I felt I couldn't go on, I would remember the doctor's encouraging words. |
|  |  | Desire for diversified health education. | P08 | I would feel a greater sense of safety and trust, and find it easier to connect with authentic peer patients, if the hospital were to organize both online and offline activities. |
|  |  |  | P12 | The medical staff have helped me a lot... I think you could teach us about this disease by phone, printed materials, or WeChat. |
| Social /Cultural-Level(Macrosystem) | Barriers to healthcare access and continuity of care. | Misdiagnosis and missed diagnosis at the primary care level. | P08 | At first, there was black discoloration of the nail with oozing, so I went to our local county hospital. The doctor diagnosed it as a viral infection... It wasn't until I went to a large hospital that it was finally diagnosed as malignant melanoma. |
|  |  |  | P09 | I noticed it last year. At that time, it was the size of a soybean. The doctor at the local hospital thought it was normal and did not perform any further examination or treatment. |
|  |  |  | P11 | I found an egg-sized lump in my armpit. After the local clinic gave me antibiotic shots, I couldn't feel it anymore, but the doctor didn't seem concerned about it. |
|  |  | Difficulty in accessing specialist resources. | P10 | After my diagnosis, I tried to find a specialist. I asked everywhere locally but couldn't find anyone. Eventually, I found this hospital online. |
|  |  | Insufficient Doctor-Patient Communication in Treatment Planning. | P06 | All I knew was that I needed surgery. I didn't know about the follow-up or complications. I wish the doctor would tell me about this disease more than once. |
|  | Heavy economic burden | The double blow of illness-induced poverty and income disruption. | P01 | I'm the only one in my family who earns money. There are so many things that need money. |
|  |  |  | P11 | We're really struggling financially. Our family has no income because of my illness. We've already spent 100,000 yuan on early treatment. |
|  | The shackles of sociocultural beliefs and their breakthrough | Disease stigmatization. | P02 | For a while after the surgery, my wound festered and oozed pus. When people saw it, they didn't dare come near me. That made me feel really terrible inside |
|  |  |  | P16 | I feel that my former friends are gradually distancing themselves from me. They probably think I have a contagious disease. |
|  |  | Digital divide and health literacy disparities. | P03 | We've been in the countryside for decades and never heard of anyone getting melanoma. I look online but don't know if the info is any good. It's really confusing. |
|  |  |  | P08 | If the hospital could organize some online and offline activities, I would feel safer and more trusting, and it would also be easier to find real fellow patients to talk with. |
|  |  |  | P09 | I don't know how to use the internet, so I hope to gain relevant knowledge through means such as telephone calls, videos, and printed handouts. |
